# Cryptotanshinone enhances the efficacy of Bcr-Abl tyrosine kinase inhibitors via inhibiting STAT3 and eIF4E signalling pathways in chronic myeloid leukaemia

**DOI:** 10.1080/13880209.2021.1944224

**Published:** 2021-07-02

**Authors:** Rubin Cheng, Yilan Huang, Yun Fang, Qirui Wang, Meixiu Yan, Yuqing Ge

**Affiliations:** a School of Pharmaceutical Sciences, Zhejiang Chinese Medical University, Hangzhou, China; b The First Affiliated Hospital of Zhejiang Chinese Medical University, Hangzhou, China

**Keywords:** Imatinib resistance, second-generation TKI, CML xenograft models, STAT3 signalling pathway

## Abstract

**Context:**

A portion of patients with chronic myeloid leukaemia (CML) develop resistance to the Bcr-Abl tyrosine kinase inhibitors (TKIs), limiting the clinical applications. Previous results have demonstrated the synergistic effects between cryptotanshinone (CPT) and imatinib on apoptosis of CML cells *in vitro*.

**Objective:**

To determine the antileukemia effects of CPT and TKIs on the resistant CML cells, and further investigate the effect of combined treatment of CPT and imatinib on tumour growth and apoptosis in the xenograft model and clarify its regulatory mechanisms.

**Materials and methods:**

The combination effects of CPT and second-generation TKIs were evaluated in resistant CML cells K562-R. CPT and imatinib were orally administered once daily for 21 days on K562-R xenografts in nude mice (6 per group). Tumour proliferation and apoptosis were examined by Ki-67, PCNA and TUNEL staining. The expression levels of apoptotic markers and activities of STAT3 and eIF4E pathways were determined via immunohistochemistry staining and western blotting analysis.

**Results:**

CPT significantly enhanced the antiproliferative effects of TKIs, via triggering cleavages of caspase proteins, and inhibiting activities of STAT3 and eIF4E pathways. The administration of CPT and imatinib dramatically inhibited the tumour growth of xenografts and achieved a suppression of 60.2%, which is 2.6-fold higher than that of single imatinib group. Furthermore, CPT and imatinib increased the apoptotic rates and markedly decreased the phosphorylation levels of STAT3 and eIF4E.

**Conclusions:**

Our results demonstrated that CPT could significantly enhance the antileukemia efficacy of TKIs, suggesting the therapeutic potential of CPT to overcome CML resistance.

## Introduction

Chronic myeloid leukaemia (CML) is a clonal stem cell disorder disease with the oncogenic fusion protein Bcr/Abl, which leads to the constitutive activation of Abl tyrosine kinase. The introduction of imatinib has significantly improved the disease management of patients in chronic phase (Jain and O'Brien 2013). However, clinical resistance to imatinib has been widely reported, particularly due to the occurrence of Bcr/Abl mutations and limited action towards patients in blast crisis (Sacha et al. 2003; Marfe and Di Stefano 2014). In addition, the significant safety issues of imatinib treatment have also been documented. Although imatinib are less toxic than traditional cytotoxic drugs, their use is still associated with various adverse reaction events and side effects. The observational study of CML management in Europe indicated that about a proportion of 31% of patients has to discontinue imatinib therapy before the complete remission due to intolerance, indicating the requirement of alternative therapy strategies (Michallet et al. 2010). Furthermore, almost 60% of patients with sustained complete molecular response relapsed within 1–2 years after the discontinuation of imatinib treatment (Thielen et al. 2013). In addition, many CML patients failed the second-generation tyrosine kinase inhibitors (TKIs) due to the Bcr/Abl dependent and independent mechanisms (El Fakih et al. 2020). Therefore, it is urgent to find more efficient therapeutic strategies to overcome these problems.

The mechanisms of resistance to TKI include mutations and amplification of Bcr/Abl fusion gene, drug efflux mediated by transporters and bone marrow microenvironment, which lead to aberrant activation of certain signalling pathways in CML cells (Beider et al. 2014; Yang and Fu 2015). It is well known that the impaired signalling pathway JAK/STAT3 and eIF4E play crucial roles in the development of imatinib resistance and CML progression. The activation of STAT3 has been implicated in the malignant transformation and closely related with drug resistance in a variety of cancers (Avalle et al. 2017). The soluble factors derived from bone marrow stromal cells protects CML cells from imatinib-mediated Bcr-Abl inhibition through STAT3 activation (Traer et al. 2012). Oroxylin A increased the sensitivity of K562 cells to imatinib by suppression of STAT3 pathway (Li et al. 2015). The combination treatment of STAT3 inhibitor and imatinib led to synthetic lethality in resistant CML cell (Eiring et al. 2017). Furthermore, the STAT3 inhibitor CDDO-Me produced synergistic growth inhibitory effects with Bcr/Abl tyrosine kinase inhibitors and provided a novel approach to overcome resistance in CML, including stem cells, highly resistant subclones with Bcr/Abl mutation T315I or T315I compound mutations (Gleixner et al. 2017). Eukaryotic translation initiation factor 4E (eIF4E) is the rate-limiting factor for cap-dependent translation initiation, a well-known mechanism that enhances the translation of specific onco-proteins involving cell cycle, apoptosis and angiogenesis regulation. Since the central roles of eIF4E in contributing tumour progression and chemotherapy resistance, it has been identified as specific indicator for poor prognosis in many types of cancer (Larsson et al. 2007). The overexpression of eIF4E also exhibited close association with imatinib resistance and disease progression in CML. The activity of eIF4E was dramatically increased in CML patients at an advanced stage. Moreover, the expression and phosphorylation of eIF4E were upregulated in a Bcr/Abl dependent manner in blast crisis CML (Yang et al. 2018). Furthermore, aberrant activation of eIF4E in leukaemia stem cells derived from blast crisis CML cells significantly increased the protein synthesis of β-catenin, acting as a reservoir for drug resistance (Lim et al. 2013). Inhibition of eIF4E exhibited synergistic effects with TKI inhibitors in CML cells, as well as reversed certain malignant phenotype (Ge et al. 2012; Shi et al. 2015). The activities of STAT3 and eIF4E pathways are spared by the TKI application and therefore could contribute to drug resistance (Stella et al. 2013; Liu et al. 2016). The combination treatment of imatinib with other agents targeting STAT3 and eIF4E pathways might an alternative therapy approach for CML patients with imatinib resistance.

Cryptotanshinone (CPT) is a quinoid diterpene extracted from the root of medicinal plant *Salvia miltiorrhiza* Bunge, which has been widely used for the treatment of various diseases. An increasing amount of research has demonstrated that CPT has pharmacological activities involving antioxidative stress, antibacteria, antiinflammation and anticancer (Hao et al. 2016; Xu et al. 2017). Our previous results have suggested the synergistic effects of CPT and imatinib on the apoptosis induction in CML cells by STAT3 and eIF4E inhibition (Ge et al. 2015a). However, the combination effects between CPT and the second-generation TKIs remain unclear. Furthermore, there is little information investigating the *in vivo* anti leukaemia effect of CPT and imatinib and its underlying mechanism. Here, we determined the effect and underlying mechanisms of combination treatment of CPT and imatinib as well as the second-generation TKIs dasatinib and nilotinib. Our results indicated that CPT efficiently cooperated with the three Bcr-Abl TKIs in both sensitive and imatinib resistance CML cells. The combination of CPT and imatinib dramatically inhibited the tumour growth and induced cell apoptosis of xenograft nude mice of imatinib resistance CML cells, suggesting that CPT could be used as a potent chemotherapeutic agent against TKI resistance in CML.

## Materials and methods

### Reagents

Cryptotanshinone (CPT) was purchased from Shanghai Yuanye Biotechnology Co., Ltd, with a purity of 98% (#B21586, Shanghai, China). High-performance liquid chromatography (HPLC) analysis was applied to further confirm the purity of CPT (98%). Imatinib (Genike) was obtained from Nanjing Chia Tai Tianqing Pharmaceutical Co., Ltd. The second-generation tyrosine kinase inhibitors nilotinib was purchased from Aladdin Chemistry Co., Ltd (#N126111, Shanghai, China). Dasatinib was obtained from Shanghai Yuanye Biotechnology Co., Ltd (#S45672, Shanghai, China). Antibodies used in this study were purchased from Cell Signalling Technology (MA, USA), including cleaved caspase-3 (#9664), cleaved PARP (#5625), p-STAT3 (#9145), Bcr-Abl (#3908), p-Bcr-Abl (#3009), p-Src (#2101) and β-Actin (#3700). Antibody for cleaved caspase-9 (Asp353) (#AF5240) was obtained from Affinity Biosciences (Cincinnati, OH, USA), for eIF4E (#610270) was obtained from BD Biosciences (San Diego, CA, USA), and p-eIF4E (ab76256) was purchased from Abcam. The rabbit polyclonal antibody against Ki-67 (PB9026) and mouse monoclonal antibody against PCNA (BM0104) was obtained from Boster Biological Technology Co., Ltd.

### Cell lines and cell culture

Human chronic myeloid leukaemia cell line K562 was purchased from the Cell Bank of Type Culture Collection of Chinese Academy of Sciences (Shanghai, China). The resistant CML cell K562-R was established and cultured according to the previous report (Ge et al. 2015a). Briefly, K562-R cells were cultured in RMPI 1640 medium containing 10% heat-inactivated foetal bovine serum, 100 U/mL penicillin, and 100 μg/mL streptomycin with a humidified 5% CO_2_ incubator at 37 °C. K562-R cells were routinely maintained in culture medium with 1 μM imatinib to keep the specific resistance to imatinib.

### Cell viability assay

The MTT assay was performed to determine cell viability according to the manufacture’s procedure. Briefly, the human CML cells K562 and K562-R were incubated with different concentrations of Bcr-Abl tyrosine kinase inhibitors (imatinib, dasatinib and nilotinib) and 10 μM CPT with a density of 1 × 10^4^ cells per well in 100 μL RPMI 1640 medium for 48 h. Then, the cells were subsequently incubated with a final concentration of 5 mg/mL MTT for 4 h and the formazan crystals were dissolved in 150 μL of dimethyl sulfoxide (DMSO). The absorbance was detected at 490 nm using a microplate reader (Thermo Fisher Scientific, Finland). The cell viability of 0 µM served as the control group.

### Tumour growth in xenografts

Four-week-old female nude mice were purchased from Zhejiang Chinese Medical University Laboratory Animal Centre (Hangzhou, China). Xenograft mouse models were established according to published protocols (Lei et al. 2018). Briefly, 4 × 10^7^ of K562-R cells were collected and implanted subcutaneously into the right flank of the nude mice. After tumour volume approximately reached the size of 80–100 mm^3^, the animals were randomised into four different groups (6 mice per group): control group (treated with 0.9% saline water only), imatinib treatment group (50 mg/kg, once daily, p.o.), CPT treatment alone (10 mg/kg, once daily, p.o.), and the combination treatment group with CPT and imatinib (10 mg/kg CPT and 50 mg/kg imatinib, once daily, p.o.). The CPT was dissolved in solution comprising 0.9% saline water, ethanol and castor oil in a 8:1:1 (v:v:v) ratio. The tumour volumes were measured every 4 days using callipers, which were determined with the following formula: V = π/6 × ab^2^ (mm^3^), where a and b represent to the longest and shortest diameters, respectively. The animal general health and toxicity were also monitored by measuring the body weight, feeding behaviour and motor activity. The drug treatment was continued for 3 weeks and tumour xenografts were immediately collected and analysed after the mice had been euthanized. All animal studies were conducted with the approval of the Institutional Animal Care and Use Committee of Zhejiang Chinese Medical University.

### HE staining and MVD analysis

The xenograft tumour tissues were immediately fixed with 4% paraformaldehyde for 72 h, and then embedded in paraffin. The HE staining was performed using standard histological techniques on the tissue specimens (5 μm thick) from each group, to determine the cell density and the necrotic area after the treatment of imatinib and CPT. The microvessel density (MVD) on tumour tissue was measured though immunohistochemical staining of CD34. The structure changes and CD34 positive staining results were observed using the optical microscope.

### TUNEL analysis

The apoptotic rates from CML tissues were analysed by Terminal deoxynucleotidyl transferase dUTP nick end labelling (TUNEL) method using the In Situ Cell Apoptosis Detection Kit I (POD) from Boster Biological Technology Co., Ltd (#MK1015). The formalin-fixed xenografts of K562-R cells were embedded in paraffin and sectioned using standard techniques. The TUNEL staining was performed and analysed according to the manufacturers' protocol. The proportions of TUNEL positive cells with brown nucleus were calculated from 5 randomly selected views under microscopic fields at ×400 magnification for each slice.

### Western blot analysis

The western blot technique was applied to analyse the protein expression in K562-R cells and tumour xenograft tissues. The K562-R cells firstly treated with the combination of 10 μM CPT and different TKIs (imatinib 2 μM; dasatinib 0.01 μM; nilotinib 0.05 μM) for 48 h. Then, K562-R cells were harvested and lysed with RIPA lysate buffer with 1 mM phenylmethylsulfonyl fluoride (PMSF) and phosphatase inhibitor. The tumour tissues were homogenised in ice-cold cell lysis buffer containing 1 mM PMSF and 1% cocktail protease inhibitors (Sigma). Protein concentrations of the extracts were measured by BCA Protein Assay and equal amount of total protein from each sample was used for subsequent experiments. The protein sample from each group was separated with SDS-PAGE and then transferred to PVDF membranes (Millipore) for about 2 h. Membranes were washed 3 times and blocked with TBST containing 5% non-fat milk for 1 h, and then the membrane was incubated with the target antibodies overnight at 4 °C, respectively. After washing with TBST three times, the membranes were incubated with anti-rabbit secondary antibody or anti-mouse secondary antibody for 1 h at room temperature. The enhanced chemiluminescence reagent (Fude Biological Technology, China) was used to detect the immunoreactive protein bands. The band intensity was further measured using Image J software. β-Actin was stained as the loading control.

### Immunohistochemistry assay

The expressions of PCNA, Ki-67, p-STAT3 and p-eIF4E on CML xenografts were assessed using immunohistochemical (IHC) staining. The tumour xenograft tissues were fixed with formalin and then embedded in paraffin and sectioned (5-μm thick) using standard techniques. The sections were immunostained with the antibodies of CD34 (dilution 1:100), PCNA (dilution 1:150), Ki-67 (dilution 1:50), p-eIF4E (dilution 1:100) and p-STAT3 (dilution 1:50) for 1–2 h at 37 °C, respectively. The PBS-washed sections were incubated with the biotinylated secondary antibody for 30 min and treated with avidin-conjugated horseradish peroxidase for another 30 min at 37 °C. The IHC staining was carried out with DAB Immunohistochemistry Colour Development Kit (#E670033) from Sangon Biotech (Shanghai) Co., Ltd based on the manufacturer’s manual. The semi-quantitative analysis of the immunostained sections was performed by Image Pro-Plus. The tissues from three individuals of each group were prepared to determine the expression levels of target proteins. Data were calculated from five randomly selected regions for each tissue section.

### Statistical analysis

Statistical analysis was done by the statistical software SPSS 17.0. The statistical significance between different groups was analysed by one-way analysis of variance (ANOVA), followed by the Tukey *post hoc* test. The values were expressed as mean ± standard deviation (SD). *p* < 0.05 was considered as statistically significant differences between different groups. All statistical tests were performed using GraphPad Prism 5.

## Results

### The antileukemia effects of CPT and Bcr-Abl tyrosine kinase inhibitors in imatinib resistant cell K562-R

**Figure 1.**
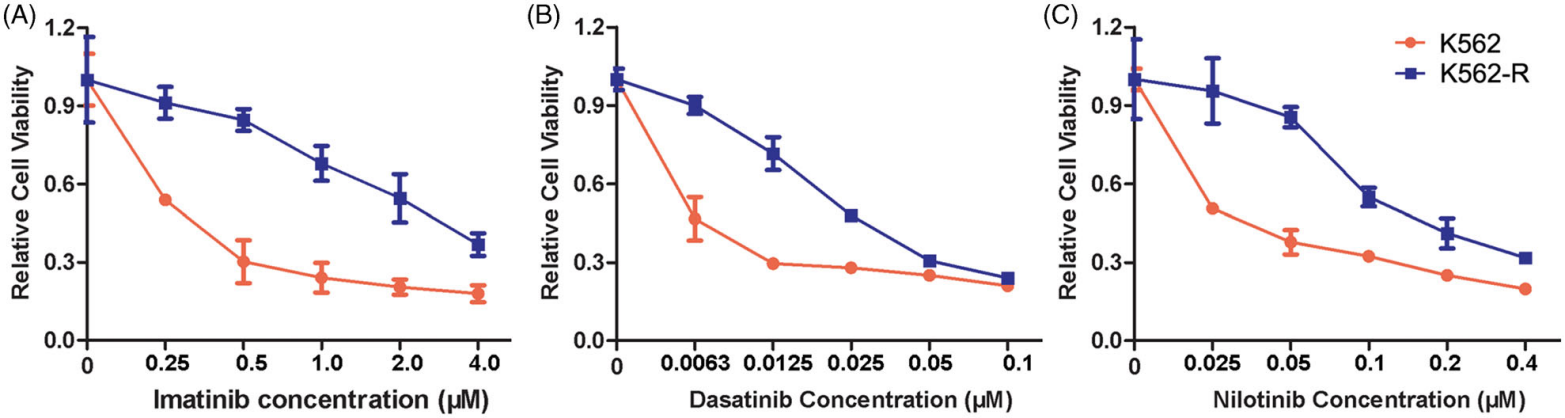
Antiproliferative effects of imatinib, dasatinib and nilotinib on the sensitive CML cell K562 and resistant CML cell K562-R. Cells were treated with different concentrations of imatinib (A), dasatinib (B) or nilotinib (C) for 48 h. The cell viability was measured using MTT assay. Data were represented as the mean ± SD obtained from three independent experiments.

In order to determine the resistance ability of different tyrosine kinase inhibitors (TKIs) in the resistant CML cells, the cell viability was investigated in both K562 and K562-R cells after the exposure of imatinib, dasatinib and nilotinib, respectively. The results confirmed the acquired imatinib resistance in K562-R cells, which was consistent with our previous reports (Figure 1(A)). Furthermore, K562-R cells exhibited obvious resistance to the second-generation TKIs dasatinib and nilotinib (Figure 1(B,C)). The treatment of 12.5 nM dasatinib induced a proliferative inhibitory rate of 70.42% in K562 cells, which was just 28.76% in K562-R cells. The IC_50_ values of imatinib, dasatinib and nilotinib on K562 cells were 0.22 μM, 2.9 nM and 22.8 nM, respectively. However, the IC_50_ values increased to 2.33 μM, 27.2 nM and 160.4 nM in K562-R cells, respectively. In addition, no point mutations were identified in Bcr-Abl tyrosine kinase domain of K562-R cells, suggesting it developed TKI resistance independent of Bcr-Abl mutations.

**Figure 2.**
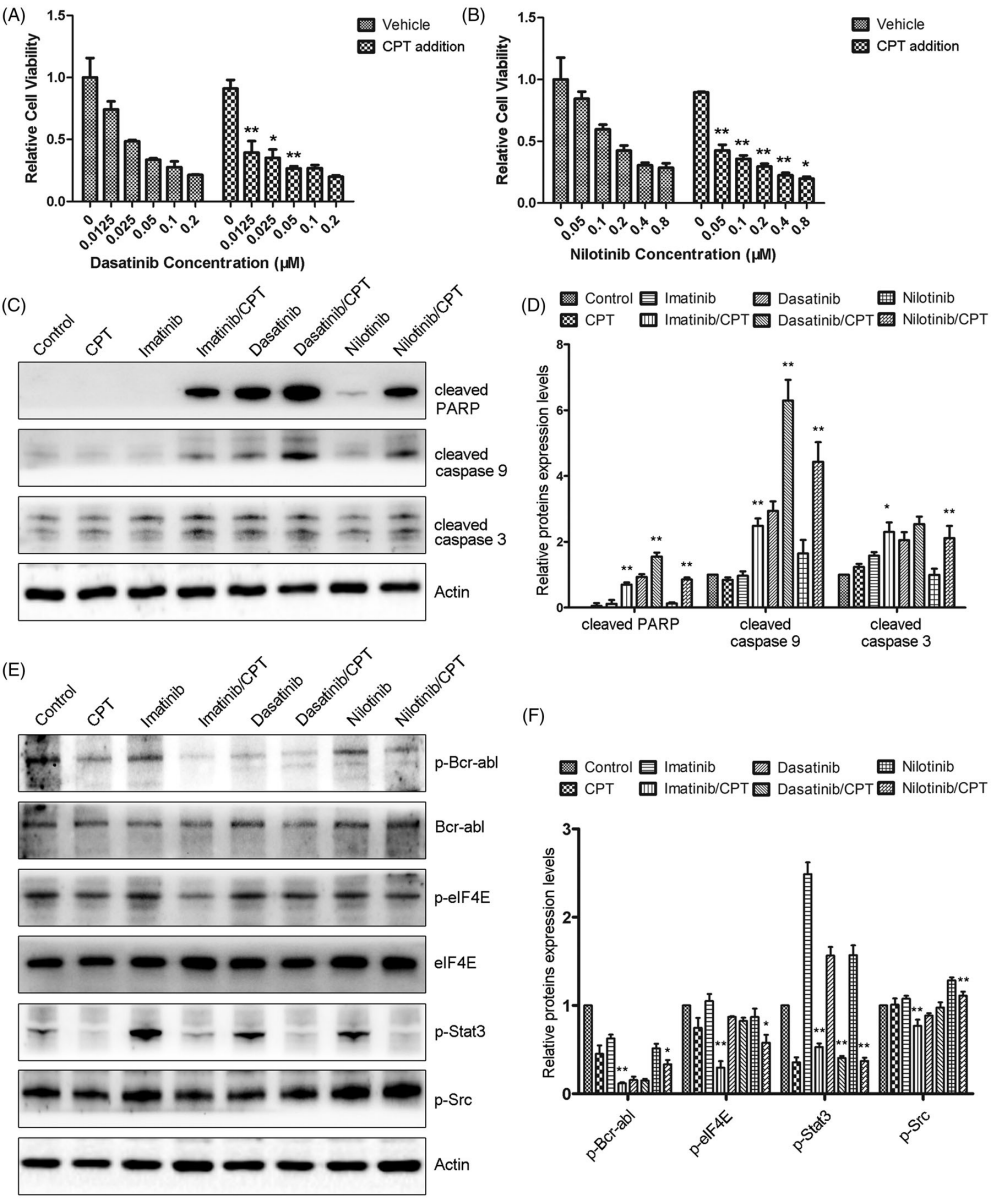
The combination effects of CPT and Bcr-Abl tyrosine kinase inhibitors on cell viability and activities of crucial apoptosis mediators in K562-R cells. (A) Cell viability of K562-R cells was determined by MTT assay after the exposure of CPT and dasatinib for 48 h. (B) The effects of CPT and nilotinib on the cell viability of K562-R cells were measured by MTT assay. Data was represented as the mean ± SD obtained from three independent experiments. (C) Protein expression of cleaved caspase-3, caspase-9 and PARP after the treatment of CPT and three TKIs were analysed by western blot. (D) The semi-quantitative immunoblotting staining analysis of the indicated apoptosis related proteins by Image J. (E) The phosphorylation and total protein expression levels of Bcr-Abl, Src, STAT3 and eIF4E were determined after the combination treatment of CPT and imatinib, dasatinib and nilotinib in K562-R cells. (F) The indicated protein expression levels were quantified by Image J. Data were expressed as the mean ± SD, n = 3. *p < 0.05, and **p < 0.01 denote significant differences compared with the single TKI treatment group.

Since the synergistic antileukemia effects of CPT and imatinib have been evaluated on K562-R cells in our previous study (Ge et al. 2015a), we further determined the combination effect and mechanism of CPT and the second-generation TKIs. The addition of CPT significantly enhanced the antiproliferative effects of dasatinib and nilotinib towards imatinib resistant CML cell K562-R (Figure 2(A,B)). The combination treatment group of CPT and 20 nM dasatinib decreased the cell proliferation of K562-R cells by 64.83%, which was much higher than the single treatment group of dasatinib (Figure 2(A)). To understand the synergistic mechanisms between CPT and TKIs, western blotting experiments were performed to determine the expression levels of apoptotic regulatory proteins in K562-R cells. Compared with the single TKIs treatment group, the combination treatment of CPT and TKI significantly increased the cleavages of pro-apoptotic protein caspase-3, caspase-9 and PARP, respectively (Figure 2(C)). The semi-quantitative analysis indicated that the cleavages of caspase-3 of nilotinib treatment group were similar with that of the control group (Figure 2(D)). While the combination treatment of CPT and nilotinib increased the protein levels of cleaved caspase-3 by 2.11-fold compared to that of the control group (Figure 2(D)). In addition, CPT enhances the inhibitory effects of TKIs on activity of Bcr-Abl kinase in the resistant CML cells (Figure 2(E)). CPT or TKIs only slightly decreased the phosphorylation of Bcr-Abl compared with the control group in K562-R. While the combination treatment of CPT and TKIs significantly decreased the expression levels of p-Bcr-Abl (Figure 2(F)). However, the phosphorylation of Src was only slightly suppressed in response to the combined treatment of CPT and TKIs (Figure 2(E)). Furthermore, CPT and three TKIs synergistically inhibited the activities of STAT3 and eIF4E signalling pathways. After the combination treatment with CPT and nilotinib, the protein levels of p-STAT3 and p-eIF4E were decreased by 66.61% and 42.14%, which was significantly lower than that of the single drug treatment group (Figure 2(F)). These results indicated that CPT promoted the antileukemia effects of TKIs through decreasing the kinase activity of Bcr-Abl, and inhibiting STAT3 and eIF4E signalling pathways in resistant CML cell K562-R.

### CPT and imatinib significantly inhibited the tumour development from the xenografted K562 resistant cells in nude mice

**Figure 3.**
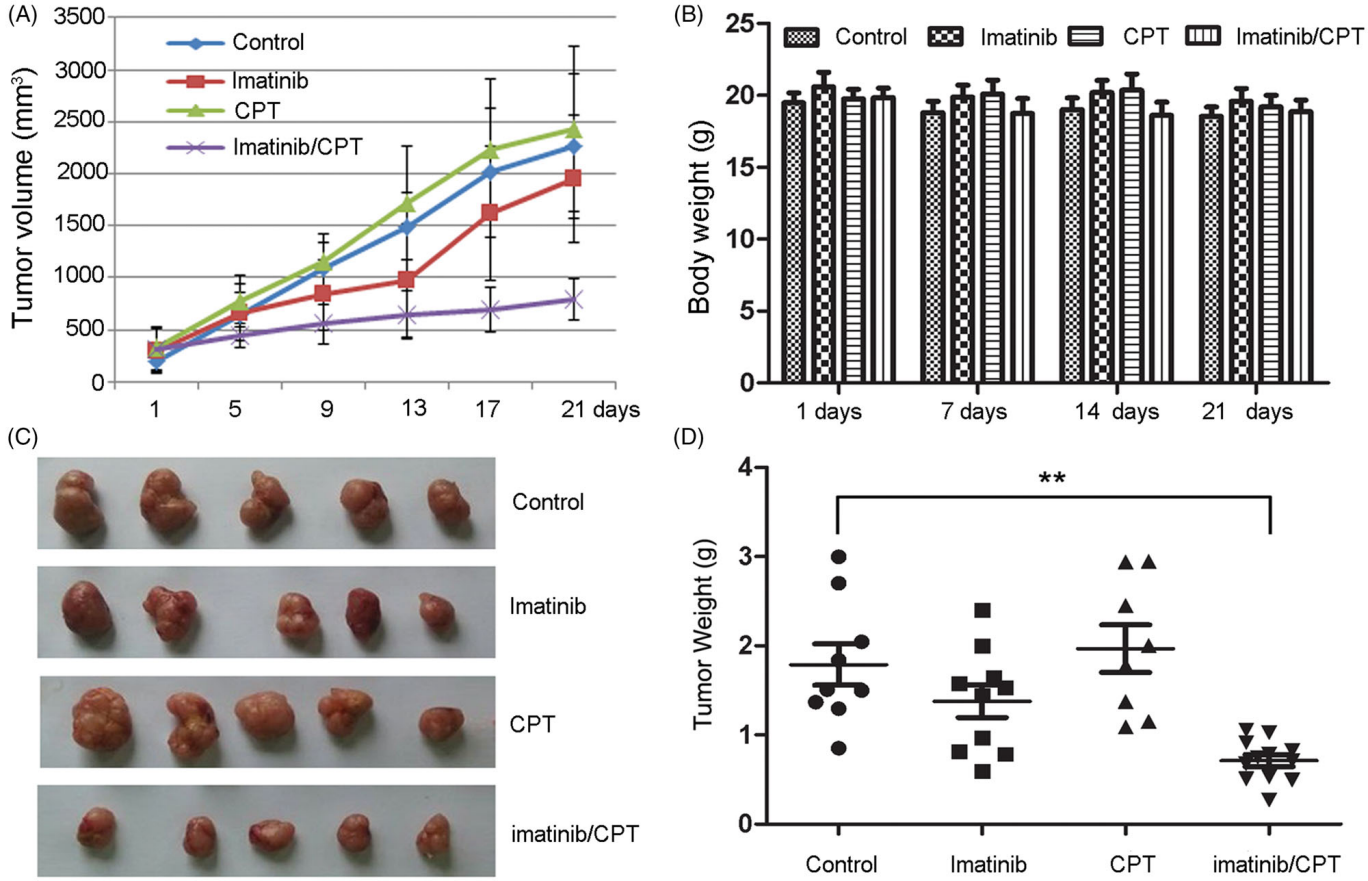
The antileukemia effect of combined imatinib and CPT on the growth of CML xenograft tumours. The imatinib resistance CML cells K562-R (4 × 10^7^ per mouse) were injected subcutaneously into the flank of nude mice. Treatment (6 mice per group) was started after the tumour volume approximately reached the size of 80–100 mm^3^. Imatinib single treatment group (50 mg/kg, daily once, p.o.), CPT single treatment group (10 mg/kg, daily once, p.o.), or the combination group of both agents. Control mice were only administrated with saline by oral gavage. (A) The average tumour volume for each group after the treatment of imatinib and CPT. (B) The body weight for each group was evaluated after the treatment of imatinib and CPT for 1 day, 7 day, 14 day and 21 day, respectively. (C) Representative images of harvested tumour tissue from four group. (D) Tumour weight for each group at the end of the observation period. The data was represented as the mean ± SD (*n* = 6). **p < 0.01 denotes significant differences compared with the control group.

To further determine the antileukemia role of CPT, we evaluated the antileukemia effects of CPT and imatinib using nude mouse xenograft model of resistant cell K562-R. A markedly smaller tumour size was noted in combination-treated mice versus that receiving vehicle alone (Figure 3(A)). While no significant differences were observed in body weight between different groups (Figure 3(B)). The appearances of harvested subcutaneous tumour and the tumour weight in each group remained consistent with the tumour size (Figure 3(C,D)). The imatinib single agent treatment partially inhibited the growth of K562-R xenograft, achieving a supspression of 14.8% in tumour size and a suppression of 23.1% in tumour weight, respectively (Figure 3). While CPT single treatment exhibited no inhibitory effect on both tumour size and tumour weight. However, CPT and imatinib combined treatment dramatically abrogated tumour growth, resulting in 64.8% reduction in tumour size and 60.2% reduction in tumour weight, respectively (Figure 3(A,D)). A significant difference was observed between single imatinib treated group and the combination treated group in tumour size and weight. Furthermore, the combination of CPT and imatinib treated mice showed no decrease in body weight or other adverse effects in behaviour and macroscopic appearance (Figure 3(B)). These results indicated that the administration of CPT and imatinib was relatively safe in the mouse model, providing potential therapeutic options for the imatinib resistant CML patients.

### CPT and imatinib induced cell apoptosis and inhibited proliferative marker expression

**Figure 4.**
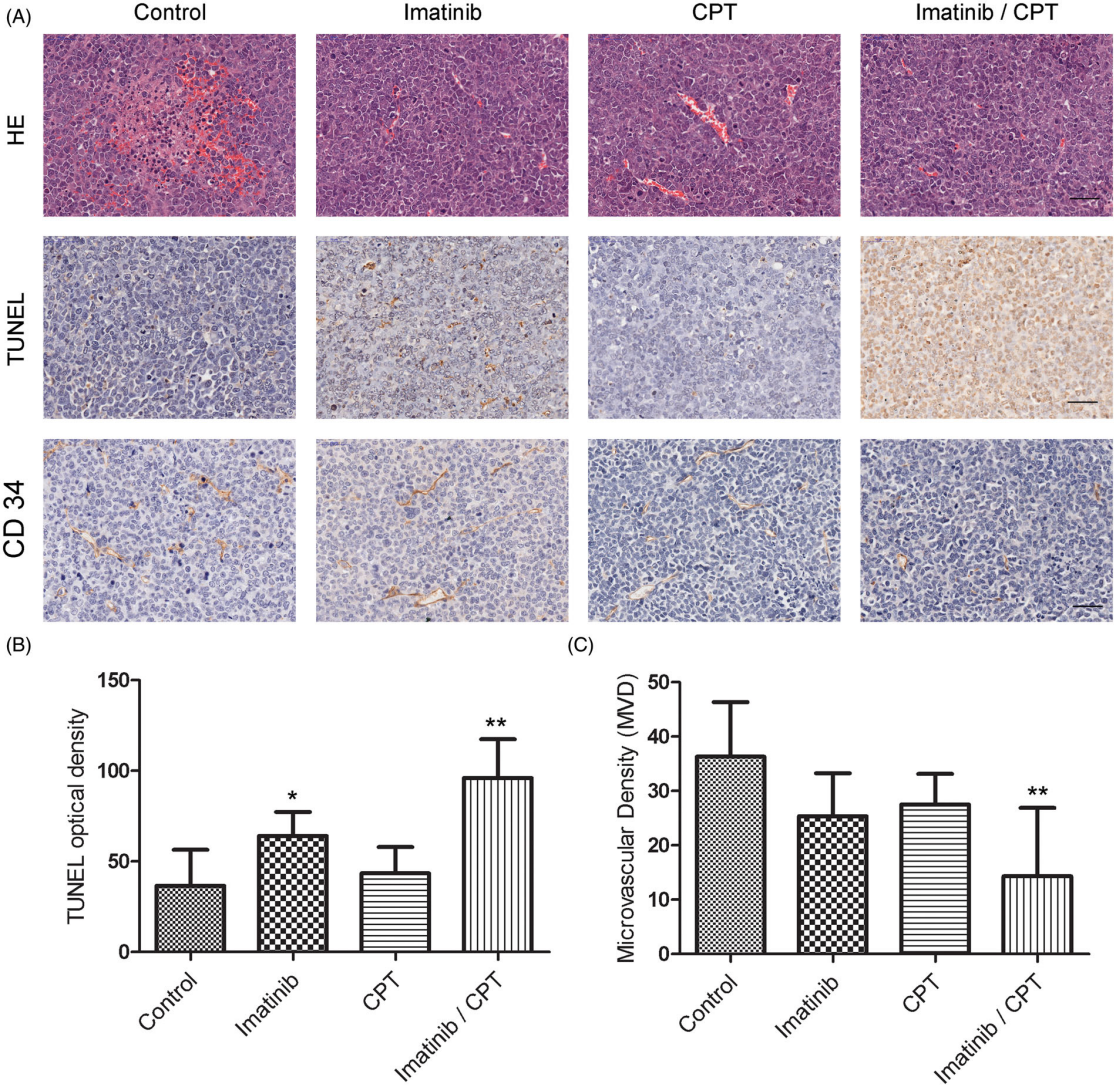
The effects of combined imatinib and CPT on the apoptotic rate and microvascular density value in tumour tissues. (A) Representative images of sections of HE staining, TUNEL staining and CD 34 staining analysis in xenograft tumour tissues produced by K562-R cells. (B) The quantified data of the apoptotic rates in tumour tissue after the treatment of imatinib and CPT. (C) The microvascular density value of each group was analysed by CD 34 staining. Scale bar = 50 µm. *p < 0.05 and **p < 0.01 denote significant differences compared with the control group.

To investigate the antileukemia effect of CPT and imatinib, we evaluated tumours for apoptosis by HE and TUNEL staining analysis. HE staining revealed a substantial increase in tumour necrosis in the combination treatment group compared to the control group (Figure 4(A)). The TUNEL positive stained cells exhibited a blue to dark blue nuclear with fragmented nuclear chromatin characteristics (Figure 4(A)). The apoptotic rate on tumour tissue in each group was determined from randomly selected image fields. As shown in Figure 4(B), tumours treated with either imatinib alone or the combination of imatinib and CPT exhibited a significantly higher percentage of positive cells than controls. In the control group, the apoptotic cell rate was only 36.6. After the treatment of single imatinib, the apoptotic cell rate increased to 64%, which was 1.75-fold higher than that of the control group. The combination of CPT and imatinib treatment group exhibited an apoptotic cell rate of 96%, which was 1.5-fold higher than that of imatinib single treatment group. However, the CPT single treated group only showed apoptosis positive cell count of 43.4%, which was just slightly higher than the control group (Figure 4(B)). Moreover, the combination treatment of CPT and imatinib significantly inhibited the degree of microvascular density (MVD) obviously on tumour tissue (Figure 4(A,C)). In the control group, the MVD value was 36.33. After the treatment of single imatinib and CPT, the MVD values decreased to 25.3 and 27.5, respectively (Figure 4(C)). Furthermore, the combination treatment of CPT and imatinib induced a MVD value of 14.3, which was much lower than that of the control group. These results suggested that the combination treatment of CPT and imatinib played antileukemia effect via apoptosis induction and angiogenesis inhibition *in vivo*.

**Figure 5.**
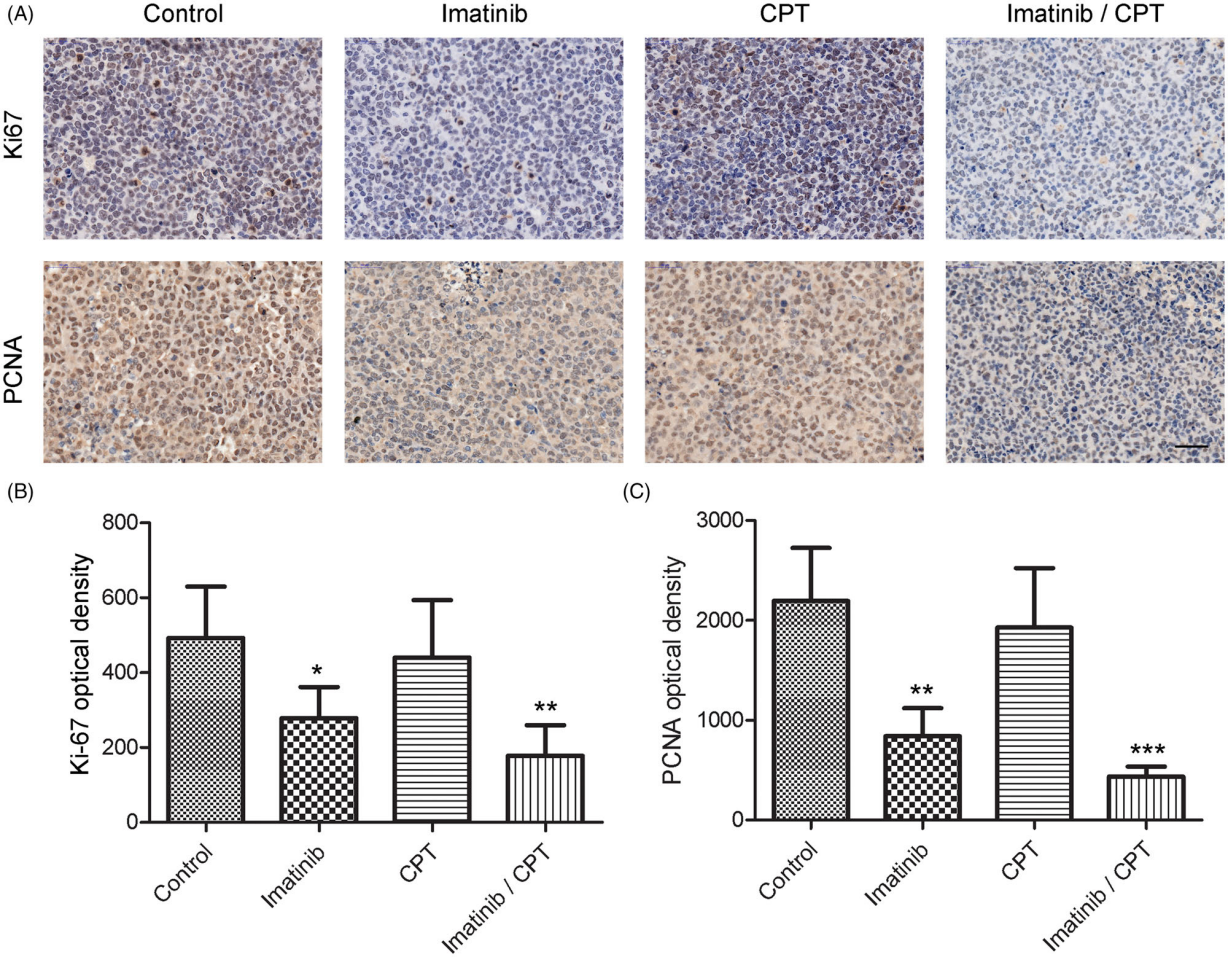
The effect of combined imatinib and CPT on the expression levels of Ki-67 and PCNA in CML xenograft tissues. (A) The expression levels of Ki-67 and PCNA were determined by immunohistochemical staining. (B) Semi-quantitative analysis of the immunohistochemical staining of Ki-67 after the treatment of imatinib and CPT. (C) Semi-quantitative analysis of the immunohistochemical staining of PCNA after the treatment of imatinib and CPT. Scale bar = 50 µm. Average data were represented as the mean ± SD (*n* = 5). *p < 0.05, **p < 0.01 and ***p < 0.001 denote significant differences compared with the control group.

To investigate the antiproliferative effects of CPT and imatinib on xenografted tumours, the expression levels of proliferative marker of Ki-67 and PCNA were determined by immunohistochemical staining. The results suggested that the expression of Ki-67 and PCNA on tumour tissues dramatically decreased in the combination treatment group compared to the control group (Figure 5(A)). The optical density value of Ki-67 was 492.38 in the control group. After the treatment of CPT and imatinib, the density value decreased to 177.58, indicating an obvious improvement on the aberrant expression of Ki-67 in xenograft tumour tissues (Figure 5(B)). Compared with the control group, the expression level of PCNA was also significantly inhibited by the combination treatment of CPT and imatinib. The PCNA optical density values were 2195.58 and 436.15 in the control group and the combination treatment group, respectively (Figure 5(C)). The combined administration of imatinib and CPT triggered a decrease on PCNA expression by 80.14% in tumour tissue (Figure 5(C)). These results demonstrated that the combination of CPT and imatinib inhibited the xenografted tumour growth of K562 resistance cells through the modulation of Ki-67 and PCNA expression *in vivo*.

### Effect of CPT and imatinib on the activities of eIF4E and STAT3 pathways

**Figure 6.**
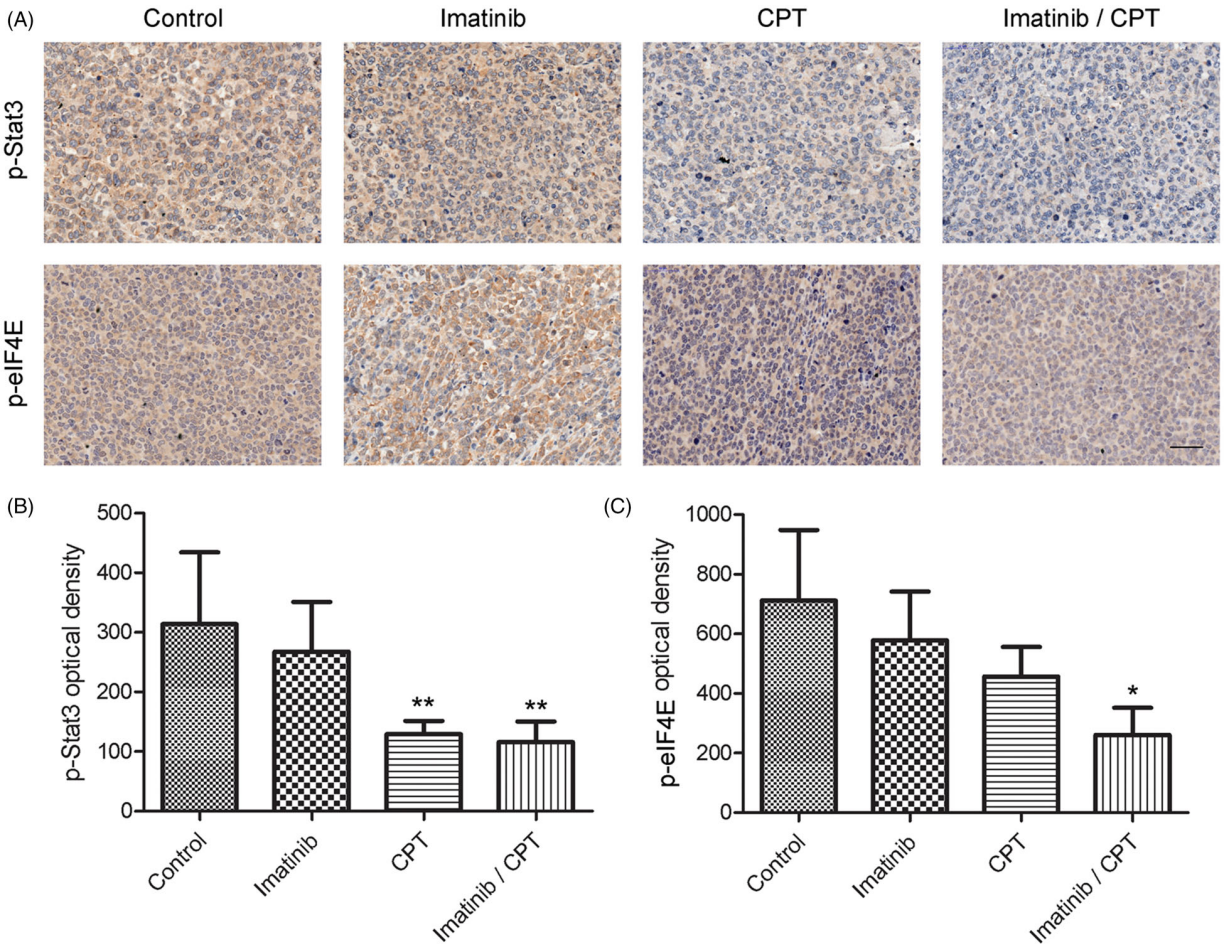
The effect of combined imatinib and CPT on the activities of STAT3 and eIF4E signalling pathway. (A) Immunohistochemistry staining of p-STAT3 and p-eIF4E on tumour tissues after the treatment of imatinib and CPT in each group. The positive staining was depicted in brown. (B) Semi-quantitative analysis of p-STAT3 staining through the image analyser. (C) Semi-quantitative analysis of the immunohistochemical staining of p-eIF4E through the image analyser. Scale bar = 50 µm. Average data were represented as the mean ± SD (*n* = 5). *p < 0.05 and **p < 0.01 denote significant differences compared with the control group.

To further understand the mechanism of combination treatment of CPT and imatinib, we analysed the changes of crucial regulators for apoptosis and proliferation in CML cell. Since the important roles of eIF4E and STAT3 signalling pathway for cell apoptosis, we determined the signalling activities after the treatment of CPT and imatinib by immunohistochemical assay and western blotting analysis. As shown in Figure 6(A), the combination treatment significantly inhibited the activities of eIF4E and STAT3 signalling pathway. The optical density values of p-STAT3 and p-eIF4E in the control group were 314.06 and 711.87, respectively (Figure 6(B,C)). After the treatment of CPT and imatinib, the positive density values of p-STAT3 and p-eIF4E decreased to 116 and 261.15, respectively, exhibiting the reductions by 63.1% and 63.4% fold compared to that of the control group. Furthermore, the CPT single treatment also significantly inhibited the activities of STAT3 and eIF4E signalling pathways in tumour tissues. Specifically, the positive cell rate of p-STAT3 was just 129.18 after CPT treatment, which was much lower than that of the control group (Figure 6(B)). In addition, imatinib single treatment showed no inhibitory effects on the expression of p-STAT3 in tumour tissues (Figure 6(B)).

**Figure 7.**
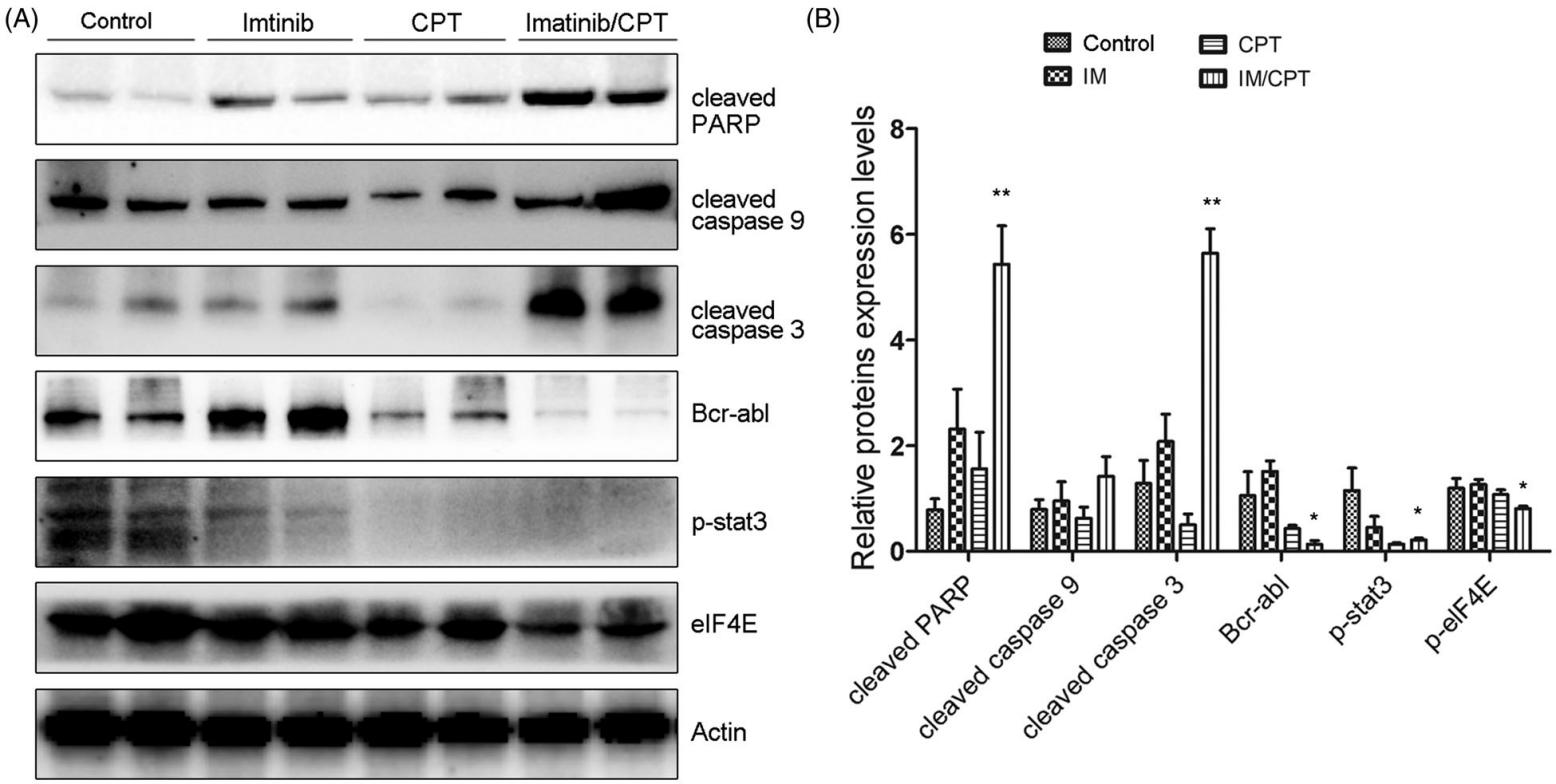
CPT and imatinib treatment promoted the cleavages of caspase proteins and inhibited the expression levels of Bcr-Abl, p-STAT3 and p-eIF4E in tumour tissues. (A) Western blotting analysis on the expression levels of cleaved caspase-3, caspase-9 and PARP of tumour tissues from different groups. (B) Quantification of the cleaved caspase proteins by Image J. (C) Western blotting analysis on the expression levels of Bcr-Abl, p-STAT3 and p-eIF4E of tumour tissues from each group. (D) Quantification of the of the indicated crucial mediators in CML. The greyscale scans analysis of the western blot images was from three independent experiments and fold changes include the normalisation to β-actin. Data are represented as the mean ± SD. *p < 0.05, and **p < 0.01 denote significant differences compared with the single imatinib treatment group.

To confirm the effect of CPT and imatinib treatment of cell apoptosis and clarify the underlining mechanisms, western blot analysis was performed to analyse the activities of crucial apoptosis mediators and STAT3 and eIF4E signalling pathways. Compared with the control group and single drug treatment group, the combination group of CPT and imatinib significantly promoted the cleavages of caspse-3, caspase-9 and PARP in tumour tissues (Figure 7(A)). Furthermore, the administration of CPT and imatinib obviously decreased the expression level of Bcr-Abl and dramatically inhibited the phosphorylation levels of STAT3 and eIF4E (Figure 7(C)). After the treatment of CPT and imatinib, the expression levels of p-STAT3 and p-eIF4E reduced by 78.62% and 19.34% fold compared to that of the control group, which was consistent with the immunohistochemical staining results (Figure 7(D)). The synergistic therapeutic effect between CPT and imatinib could be attribute to the decreased activity of STAT3 and eIF4E pathways. Overall, these results demonstrated that the combination of CPT and imatinib decreased the tumour growth and induced cell apoptosis through eIF4E and STAT3 inhibition *in vivo*.

## Discussion

In this study, we investigate the antileukemia effect of CPT and imatinib as well as second-generation Bcr-Abl tyrosine kinase inhibitors. Our results demonstrated that CPT could enhance the antiproliferative and apoptosis induction efficiency of TKIs in resistant CML cells. In addition, CPT significantly improved the inhibitory effect of imatinib towards xenografted tumour by K562 resistant cells. the combination treatment of CPT and imatinib induced cell proliferation inhibition and apoptosis by decreasing the expression levels of Ki-67 and PCNA, and reducing the activities of STAT3 and eIF4E signalling pathways *in vivo*. These results provide additional evidence for the development of therapeutic strategy through combination treatment of CPT and imatinib for chronic myeloid leukaemia patients resistant to imatinib.

Despite the high efficiency of imatinib as a single agent, the emergence of resistance to the monotherapy has commonly occurs in CML patients, especially those in advanced stages (Lim et al. 2013). The well documented mechanisms of acquired imatinib resistance include Bcr/Abl kinase domain point mutations, genomic amplification of Bcr/Abl gene and additional chromosomal aberrations (Peng et al. 2012). In particular, the T315I point mutation is the predominant mutation impairing the drug binding cluster region, which could lead to imatinib resistance and even maintain strong resistance towards the approved second-generation Bcr/Abl TKIs (Cortes et al. 2007). The long noncoding RNA HOTAIR was reported to also play important roles in mediating imatinib resistance in CML cells (Wang et al. 2017). Furthermore, the bone marrow microenvironment also confers imatinib resistance to CML through increasing STAT3 phosphorylation and enhancing the expressions of antiapoptotic proteins and P-glycoprotein (Li et al. 2015). Previous reports indicated daily oral administration of 25 mg/kg imatinib failed to inhibit the growth of xenografted KBM5 cells with T315I mutation in nude mice (Lei et al. 2018). While, imatinib treatment at concentrations of 50 mg/kg and 100 mg/kg twice daily exhibited obvious antitumor activity in GIST PDX models (Gebreyohannes et al. 2019). Our results indicated that single imatinib treatment with 50 mg/kg once daily reduced the tumour weight of K562-R xenografts by only 23.1% (Figure 3(D)). The combination treatment of imatinib along with other alternative drugs is a feasible and promising approach to overcome CML drug resistance (Beider et al. 2014). Cryptotanshinone has exhibited significant anticancer actions against different cancers by inhibiting cell proliferation, modulating cell cycle arrest, activating cell apoptosis and autophagic cell death, and deducting tumour angiogenesis (Zhu et al. 2016; Xu et al. 2017). Our previous results indicated that CPT increased the antileukemia effect of imatinib in a Bcr/Abl dependent manner *in vitro* (Ge et al. 2015b). In this study, we demonstrated that the combination of CPT and imatinib treatment synergistically induced tumour growth suppression in xenograft models produced by CML resistant cells. These results further indicated that CPT could be developed as a potential chemotherapeutic agent for the treatment of imatinib resistant CML patients.

The Ki67 and PCNA proteins are standard proliferative makers that are commonly used to assess the growth fraction of cancer cell populations (Juríková et al. 2016). Out results demonstrated the combination therapy by imatinib and CPT significantly decreased the two proliferative indicators in xenograft tissues (Figure 5). The expression of PCNA and Ki67 has been identified as a significant predictor for the development of lymph node metastases and tumour progression in colorectal cancer, which closely correlated with the representative antiapoptotic protein Bcl-2 (Guzińska-Ustymowicz et al. 2009). Moreover, the novel regulator miR-638 of leukemic cells could promote proliferation and contribute to the transformation by reduction the expression of PCNA (Lin et al. 2015). In the present study, we found that CPT significantly enhanced the inhibitory effect of imatinib on the expressions of both PCNA and Ki67 proteins. Meanwhile, the apoptotic rate of combination therapy was also significantly higher than that of imatinib single treatment group in CML xenograft. These observations indicated that CPT could dramatically enhance the anticancer effect of imatinib by proliferation inhibition and apoptosis induction *in vivo*, which was consistent with our previous findings (Ge et al. 2015a).

Resistance to imatinib and other TKIs has emerged as a major clinical problem. The STAT3 signalling pathways have been indicated to contribute to the development of imatinib resistance in CML cells through both Bcr/Abl dependent and independent mechanisms. The STAT3 pathway was a crucial mediator between the bone marrow microenvironment and minimal residual CML disease (Kuepper et al. 2019). The STAT3 inhibitor BP-5-087 targeting the SH2 domain could restore the TKI sensitivity to therapy-resistant CML progenitor cells. The combined STAT3 inhibitor and tyrosine kinase inhibitor induced synthetic lethality in therapy-resistant CML (Eiring et al. 2017). CPT has been reported to be an effective STAT3 inhibitor via binding to the SH2 domain in prostate cancer cells (Shin et al. 2009). Our previous results also demonstrated that CPT exhibited proliferation suppression and apoptosis induction effect towards pancreatic cancer cells through inhibiting STAT3 signalling activity (Ge et al. 2015b). In this study, we found CPT could significantly decrease the expression of phosphorylation of STAT3 in CML xenograft model. In addition, the activity of eIF4E signalling pathway was obviously inhibited after the treatment of CPT, further increasing the antileukemia effect of imatinib *in vivo*. Our results demonstrated that STAT3 and eIF4E pathway may be important molecular mechanisms by which CPT exhibited synergistic activity with imatinib against CML.

The decreased MVD as well as lower degree of angiogenesis have served as predictor for positive clinical outcome for TKI therapy in CML patients (Ćojbašić et al. 2017). Out results demonstrated that decreased MVD in xenograft tissue by CPT dramatically enhanced the antileukemia effect of imatinib, suggesting further investigation of active agents targeting MVD and tumour angiogenesis in CML. Although CPT has shown obvious cell cycle arrest and apoptosis induction effect towards multidrug resistant CML cells *in vitro*, the CPT single treatment group exhibited slightly inhibition on the tumour growth in CML xenograft model (Figure 3). The poor inhibitory effect of single CPT treatment could be partially attributed to inefficiency on apoptosis induction and Ki-67/PCNA expression inhibition in xenograft tissue (Figure 5). The poor aqueous solubility and oral bioavailability of CPT may also limit its bioactivities and physicochemical properties *in vivo*. The oral administration of CPT was reported to be distributed rapidly and widely in rats, with the highest concentrations in liver, lung and kidney (Wang et al. 2020). Furthermore, the plasma concentration of CPT was remarkably enhanced in rats receiving the liposoluble extract from *Salvia miltiorrhiza* comparing with that receiving pure compound, providing potential strategy to increase the antitumor effects of CPT (Wang et al. 2020). However, both of the dosage and approach of CPT administration as well as the CML mouse modelling method need to be improved to further confirm the antileukemia effect of CPT *in vivo*.

In conclusion, we have demonstrated the synergistic antileukemia activity of CPT and TKIs in resistant CML cells. Furthermore, CPT and imatinib treatment significantly inhibited tumour growth of imatinib resistant K562 cells *in vivo* through suppression of STAT3 and eIF4E signalling pathways. This result provides a new approach for the treatment of antileukemia therapy with TKI resistance.
